# Editorial Department

**Published:** 1885-04

**Authors:** 


### The Bistoury.

ELMIRA, N. Y., APRIL 1885.

The BISTOURY is published Quarterly, upon the first of April, July, October and January, at Fifty Cents a year in advance.

### Editorial Department.

BYE BYE.—When this number of the BISTOURY reaches our subscribers, the editor hopes to have escaped to a less wintry clime. In our next number we will tell you how the fishing is in the Bermuda Islands.

COCAINE.—Oh! say, *do* "give us a rest" on extolling the virtues of cocaine. We have stood more than a reasonable amount of the laudation of that most excellent drug, for an en[illegible]re year. We recognize all of its virtues, b[illegible]ieve more than one-half that is written of its m[illegible]rvelous effects, and are willing to believe that it will subdue pain anywhere, prevent pain anywhere else, and stupefy anything or anybody but the chap who wants to write about it. Now, "let up" for a while.

MICROBIOLOGY.—This is a new word coined to meet the requirements of the searchers after microbes. Since Koch's investigations of these minute organisms, which caused all the scientists to renew their studies to determine the part that bacteria perform in certain diseases, the Germans have taken so much interest in these investigations that they have established [illegible] chair of microbiology in one of their Universities, raising the same to the dignity of a recognized science. Of course, there is much objection raised, by various scientists, to recognizing Koch's cholera bacillus; but since most of these objectors agree that there is a special bacterium to be found in the "hoof disease" of animals, in erysipelas, in yellow fever, in scarlet fever, in small pox and in some other contagious and infectious diseases, it seems very probable and altogether reasonable that cholera possesses its special germ. Much remains to be learned with regard to the germ-theory of disease, and we may reasonably hope that the new chair of microbiology will be instrumental in clearing away the doubts with which the new science is surrounded.

THE DEER HOUNING BILL.—It has passed the Assembly and doubtless will the Senate. Hope Governor Hill will speedily make it a law.

A LULLABY.—The New Orleans *Times Democrat* announces that at the press dinner given by Commissioner Truman, Mr. E. L. Adams of this city, was called upon to respond to a toast, which he did in a highly entertaining manner, singing a Swedish Lullaby at the termination. Mr. Adams is well known at home as a witty speech maker, an excellent story teller, and a charming singer. But that lullaby must have been particularly well rendered since Mr. Adams has been practicing for the past three months, during every night, with all manner of lullaby songs. He has so charmed his own baby with his rendition of lullabys that it sleeps all day long that it may be up during the night to listen to the lullaby business. Yes, we would gladly have listened to that New Orleans lullaby.

MILK AS A VEHICLE OF DISEASE.—Cow's milk has been suspected for a long while as a possible means for conveying infectious and epidemic diseases from one household to another. Dr. Ballard, of Islington, as long ago as 1870 published an account of more than one hundred cases of typhoid fever directly traced to milk which had been distributed from a farm where the fever prevailed. That scarlet fever has been so transmitted, will not admit of a doubt, since numerous well authenticated cases have, from time to time, been published in the various medical journals throughout the land. Milk is not only capable of transmitting the germs of various contagious and infectious diseases, but has been found to be an excellent vehicle in which the specific microbes reproduce themselves—a "culture fluid," in fact. Then, milk often contains a fungus—the *pencillium*, when not kept in a cleanly and cool place, that frequently occasions an inflammation of the mucus membrane of stomach and bowel. An inflamed udder of the cow will yield milk that inflames the mouth of the "bottle-bed" infant, often causing serious consequences. The so-called "milk disease" which has been so prevalent in the western states, and, concerning which so much was written a few years since, was found to be the result of the animals feeding upon the poisonous *rhus toxicodendron*. So, it will be seen that milk is capable of absorbing, propagating and conveying germs of disease, and that we should exert great care in the selection of our milk-purveyor and that he, in turn, should guard his cattle against possible danger from poisonous plants and impure water, storing the milk in a suitable house, remote from the dwelling where the germs of infectious diseases are not likely to enter.

---

BLATANT BLATHERSKITES.—Are we not extending a trifle too much "freedom of speech" to our adopted fellow citizens? When Michael Schwab is permitted to travel from city to city, collecting immense crowds of people and addressing them in the most incendiary manner—urging them to deeds which, if performed, would cause his followers to be cast into prison and many of them to pay the penalty for murder; when he urges the lower and degraded classes, to which he belongs, to raise in their might to disposess the rich man of his earnings that it may be distributed among his lazy, drunken, brutal followers—has he not just a little too much freedom of speech and action?

Then, there is that dynamite fiend—O'Donovan Rossa—advocating the destruction of innocent women and children by an explosive that he would be too cowardly to approach were *he* selected to do the firing. At a safe distance, he writes his bloodthirsty epistles, urges that others blow up public buildings and ocean steamers, destroying innocent lives and much valuable property, pretending he believes that, in any possible way, Ireland could be benefitted thereby.

It is not that any body or anything is endangered by these blatherskites. These be not the recommendations of brave or wise men. But they *do* sometimes succeed in misleading their breathern abroad into performing acts which themselves would not dare to approach.

Mrs. Dudley succeeded admirably in revealing the true character of one of these miserable cowards when she stuck a pin in the back of one of them frightening him into believing that he had been shot and inducing him to make an ante-mortum statement that the entire English army had landed in America to take his miserably puny life.

Of such stuff are these braggarts made. They do not merit sufficient attention to have them distinguished in being suppressed, yet they are given too much freedom in being permitted to nauseate respectable and law-abiding people. If, every time one of them is encountered upon the street, they were kicked out of the way and little boys were taught to fire pop-guns at them, they would keep more closely to their hiding-places, thus relieving a long suffering and patient people of their presence.

---

EXPERIMENTAL MEDICATION.—No mortal probably, was ever mildly or dangerously sick that did not have an abundance of pseudo-medical advice from kind and meddlesome friends, who, either insisted that the patient partake of some favorite decoction or mixture, or try the supposed skill of an illiterate adventurer that proclaimed himself a "doctor."

The regular physician recognizes the fact that, though two or more individuals may be attacked with the same malady, yet must he vary and modify his treatment to accommodate the peculiarities of temperment or idocyncracies of each individual patient, which are likely to modify the effects of the remedial agents usuallv employed in the management of the particular disease in hand. He knows that one patient, when in health, can eat and digest a certain food that will surely poison and make sick the other. In like manner has he found that the medicant that will relieve the one will augment the distressing symptoms of the other. So, the intelligct physician, having acquainted himself thoroughly with the action of drugs and the significance of symptoms in the sick, is able, through long experience and experimentation, to bring the disease which he seeks to control to a successful issue. Not so with the layman or the ignorant pretender to skill in the practice of medicine. They have found that a certain drug, or a special method relieved, or *thought* it did, an acquaintance of an ailment the symptoms of which, they think, correspond to that exhibited in another subject whom they have unfortunately encountered. They lose no time therefore, in recommending this favorite nostrum or pretended "doctor" to the suffering man, often, honestly and sincerely believing that a "certain cure" will ensue should their recommendation of ways and means be adopted. It is no discouragement to them if they are informed that many learned and notably skillful and eminent medical men have pronounced the disease incurable. The knowledge of a learned specialist, who has devoted a life-time to the study and investigation of cancer, for instance, who can, by the aid of his microscope, name the form of cancer from the appearance of the cancer-cell, and definately tell you whether the one under observation is curable or not, counts for nothing when the ignorant "cancer-doctor," who cures with "roots and yarbs," invades the sick-room and promises a "sure cure" to the suffering invalid. He will talk very self-satisfactorily about the "rose" and "spider" cancer. Tell how his plaster will "eat out the cancer," destroy its roots and leave the uncancerous portion sound and untouched. And, strange to contemplate, many will believe him while they suffer the tortures of the damned until death comes to their relief, rather than rely upon the advice of a capable and honest physician.

Fortunately, now and then, these free-advice meddlers with their cancer-doctors, Indian doctors, mind-cure doctors, laying-on-of-hands doctors *et id omne genus*, meet with rebuff from the hands of intelligent sick people whose common sense does not desert them in sickness and threatened death. We have an eminent example of this sort of fortitude in that old warrior, General Grant. It is well known throughout the entire Nation that the General is sadly afflicted with a cancer of the throat and tongue. He called in eminent and able advice. Four of as able physicians as New York affords, pronounced his case one of cancer, and furthermore, gave it as their unanimous opinion that it is incurable. The brave, old General, nothing daunted, set about to make himself as comfortable as possible, while he tries to complete his reminiscences of the war upon which he was engaged when attacked by the fatal disease. Meanwhile, he has not escaped the entreaties of those well-meaning but meddlesome bodies who think they know of one of those ubiquitous "cancer doctors" who, in their opinion, *has* cured and doubtless *can* cure the General's cancer. It is a comfort to know that, notwithstanding the supposed famous doctor was surreptitiously introduced into his house, the General stoutly refused him admittance to his bed-chamber. He trusts implicitly in his able medical attendants, believing that if their skill cannot baffle the inroads of the disease, such a desideratum will not be found at the hands of an ignorant cancer-doctor. The physicians in attendance upon the General made no objection to the admission of the "cancer-doctor" provided he would submit to them the method of his treatment, that they might determine whether it was a safe one or even possessed any merit whatever. This proposition, as is the custom with quacks, was stoutly refused, whereupon the "distinguished specialist" was excused to go in search of a more easily duped patient.

Were all such impostors as summarily treated as in this instance, a deal of suffering would be spared the innumerable unfortunates throughout our land who are beguiled into a so-called treatment that only brings torture and a horrible death.

---

MEDICAL RESEARCH IN THE UNITED STATES.— We wonder whether it has ever occurred to our Members of Congress that this great Nation of ours has not contributed anything or exhibited the slightest interest in a department of science to which other Nations (notably Germany, France and England) have given much timely aid? We refer to medical research. Is it not passing strange that our government should appropriate millions of dollars to the investigation and construction of death-dealing contrivances and remain wholly inactive and careless as to the desirability of securing a better knowledge of methods for promoting life and health?

All investigations and discoveries that have transpired in recent times, whereby much valuable knowledge has been gleaned with regard to the causes, prevention and cure of disease, has been done under the patronage of foreign governments, while this country has been content to look idly on, availing itself of the benefits of such investigations without contributing of its abundant means the slightest aid in furtherance of the cause! Is not this inaction shameful?

Certainly, the United States contain medical gentlemen as skillful, as able and as willing to enter this field for investigation as do the countries referred to. But, such studies are costly, and very unremunerative. Scientists who possess the ability for such an undertaking, are proverbally without the requisite means to support themselves or to supply apparatus while the non-money-making employment is going on. In order that the investigators may be thoroughly untrammelled and unhindered, it becomes at once apparent that the necessities of life must be freely furnished. This, the government should freely and fully extend to them.

We see no good reason why Congress should not appropriate from ten to twenty thousand dollars yearly, for the use of the Surgeon General's office, to be devoted exclusively to medical research. This certainly would be considered a very small sum for so very important an undertaking and concerning which not only the Nation, but humanity in general is most thoroughly interested.

We hope that our legislators, among whom exist many medical men, will bring the attention of Congress to the importance of this subject.

"VEXATIOUS."—A mild term exasperatingly inscribed to the deceptive contrivance known as the fountain pen. We have confidingly and expectantly invested in several varieties of them before we fully satisfied ourself that they are an invention of his sulphurious majesty, who adapted them especially for inditing swear-words. To no other construction of phrases will it readily respond. It is not safe therefore for, nor do we advise it to be used by, a minister or an editor of prayer-books.

Should St. Peter attempt the use of one in the celestial regions, his seat as Recorder would be vacated the first day. No company of saints would endure the enforced profanity of the old man.

If any chap has ever succeeded in using any sort of a fountain pen satifactorily, and is sane, and can give satisfactory references for veracity, and will send us his photograph with a glory-hoop about his head, we will give the same a place in our sanctum.

CREMATION.—We have at various times, in the columns of the BISTOURY, pointed out the great danger to which the living are subjected through the persistency of an otherwise intelligent people in burying their dead. Nothing that we can at this time add will give the subject any more importance than we have heretofore striven to make manifest. We desire only to point out the increased danger that surrounds the system of hiding dead bodies in the ground if we should be visited, this summer with an epidemic of cholera. All scientists agree that our opportunities for escaping this much dreaded disease are greatly enhanced by the observance of alsolute cleanliness of person and premises and the procurement of healthful water.

No city, the size of Elmira can secure potable water from wells. The decomposing animal and vegetable substances—the natural offal from dwellings, the sewage from cess-pools and from water closets that surely find their way into all wells, make the drinking water from this source, absolutely dangerous. To this form of pollution is also added that which is afforded by the cemeteries. Located, as they usually are, upon a hill-side, it becomes an easy matter for the rain to find the decomposing body under the loosened earth, washing the disease-producing germs to the valley below and into the drinking fountains.

Manifestly, the remedy for all this is *not* to use water from wells, and, in order to spare the river and other water supplies from the pollution of the grave-yards, cremate the dead.

Upon the cleanliness, beauty, and wholesomeness of this procedure we have heretofore fully commented.

